# The interaction between freezing tolerance and phenology in temperate deciduous trees

**DOI:** 10.3389/fpls.2014.00541

**Published:** 2014-10-10

**Authors:** Yann Vitasse, Armando Lenz, Christian Körner

**Affiliations:** Institute of Botany, University of BaselBasel, Switzerland

**Keywords:** biogeographical limits, cold acclimation, freezing resistance, fundamental niche, leaf-out, phenology, plant–climate interactions, temperate trees

## Abstract

Temperate climates are defined by distinct temperature seasonality with large and often unpredictable weather during any of the four seasons. To thrive in such climates, trees have to withstand a cold winter and the stochastic occurrence of freeze events during any time of the year. The physiological mechanisms trees adopt to escape, avoid, and tolerate freezing temperatures include a cold acclimation in autumn, a dormancy period during winter (leafless in deciduous trees), and the maintenance of a certain freezing tolerance during dehardening in early spring. The change from one phase to the next is mediated by complex interactions between temperature and photoperiod. This review aims at providing an overview of the interplay between phenology of leaves and species-specific freezing resistance. First, we address the long-term evolutionary responses that enabled temperate trees to tolerate certain low temperature extremes. We provide evidence that short term acclimation of freezing resistance plays a crucial role both in dormant and active buds, including re-acclimation to cold conditions following warm spells. This ability declines to almost zero during leaf emergence. Second, we show that the risk that native temperate trees encounter freeze injuries is low and is confined to spring and underline that this risk might be altered by climate warming depending on species-specific phenological responses to environmental cues.

## INTRODUCTION

Since approximately 11,000 years, temperate trees have evolved into an interglacial period, the Holocene, which is warmer than the preceding 100,000 years, i.e., the last glacial period. While the Holocene is climatically stable when viewed from a geological time perspective, the climate of temperate areas is highly variable from the perspective of a trees’ lifespan, yet with a predictable seasonality. As a result, temperate trees have evolved to cope with the seasonal occurrence of freezing temperature. One part of the adaptation to seasonality is cold acclimation in autumn and a dormancy period in winter, followed by a deacclimation period in spring. The initiation and the progress of the acclimation and deacclimation periods and the onset of growth are mediated by temperature and photoperiod (Polgar and Primack, 2011). A good match between these physiological processes and climatic conditions is essential for the persistence of trees in temperate areas.

The term phenology is commonly used to refer to the timing of the seasonal development in plants, most commonly, the visible changes in plant appearance such as greening in spring, flowering, and senescence. In deciduous trees growing in temperate climates, the timing of phenological events affects annual growth, reproductive success, and competitive abilities of trees (Myneni et al., 1997; Hasenauer et al., 1999; Chuine and Beaubien, 2001; Chuine, 2010). Thus, phenology has a major impact on fitness and distribution of tree species. Currently, global warming is modifying the phenology of temperate trees, particularly the onset of spring, in Europe (e.g., Menzel, 2000; Menzel et al., 2006; Fu et al., 2014b; Kolářová et al., 2014), North America (e.g., Beaubien and Freeland, 2000; Schwartz and Reiter, 2000) and Eurasia (e.g., Chen et al., 2005; Ma and Zhou, 2012). Changes in phenology may even feed back on climate (Richardson et al., 2013). Since about two decades, there is a renewed interest in the overall impact of phenological shifts in forest ecosystems due to climate change. It has recently led to substantial advances and new perspectives in the understanding of the environmental factors driving tree phenology (e.g., Körner and Basler, 2010; Basler and Körner, 2012; Fu et al., 2013; Dantec et al., 2014; Laube et al., 2014; Polgar et al., 2014). Concurrent with the shift in phenology, the risk of trees to encounter freezing damages is changing, yet it is still unclear whether the risk increases or not (Hänninen, 1991; Taulavuori et al., 2004; Augspurger, 2009; Bennie et al., 2010; Hänninen and Tanino, 2011).

Here we aim at providing an overview of the interplay between phenology, species-specific freezing resistance and low temperature range limits of temperate trees. Instead of sequentially reviewing the annual phenological cycle of temperate trees, we emphasize the interaction between tree phenology and seasonal climate in an evolutionary context. Thus, we will highlight the role of seasonal variations in climate as the main evolutionary pressure that has led deciduous trees to develop long-term adaptations and rapid responses to escape or tolerate freezing temperatures in temperate areas. We will first focus on the winter dormancy period, which can be considered as a long-term evolutionary response of temperate trees to tolerate extreme low temperatures. We further will summarize knowledge on genetic adaptation of phenology and cold hardiness developed by tree populations living at the cold edge of their distribution range. Next, we will show the ability of trees to rapidly modify their cold hardiness and phenology in response to climatic fluctuations. In particular, we discuss to what extent dormant and active buds are able to rapidly acclimate to colder temperature. Finally we will discuss which period of the year is the most critical in terms of the risk to encounter freezing damages and how this risk could change with climate warming.

## SEASONALITY IN TEMPERATE CLIMATE

### SEASONAL CHANGES OF TEMPERATURE AND PHOTOPERIOD

**FIGURE 1:**
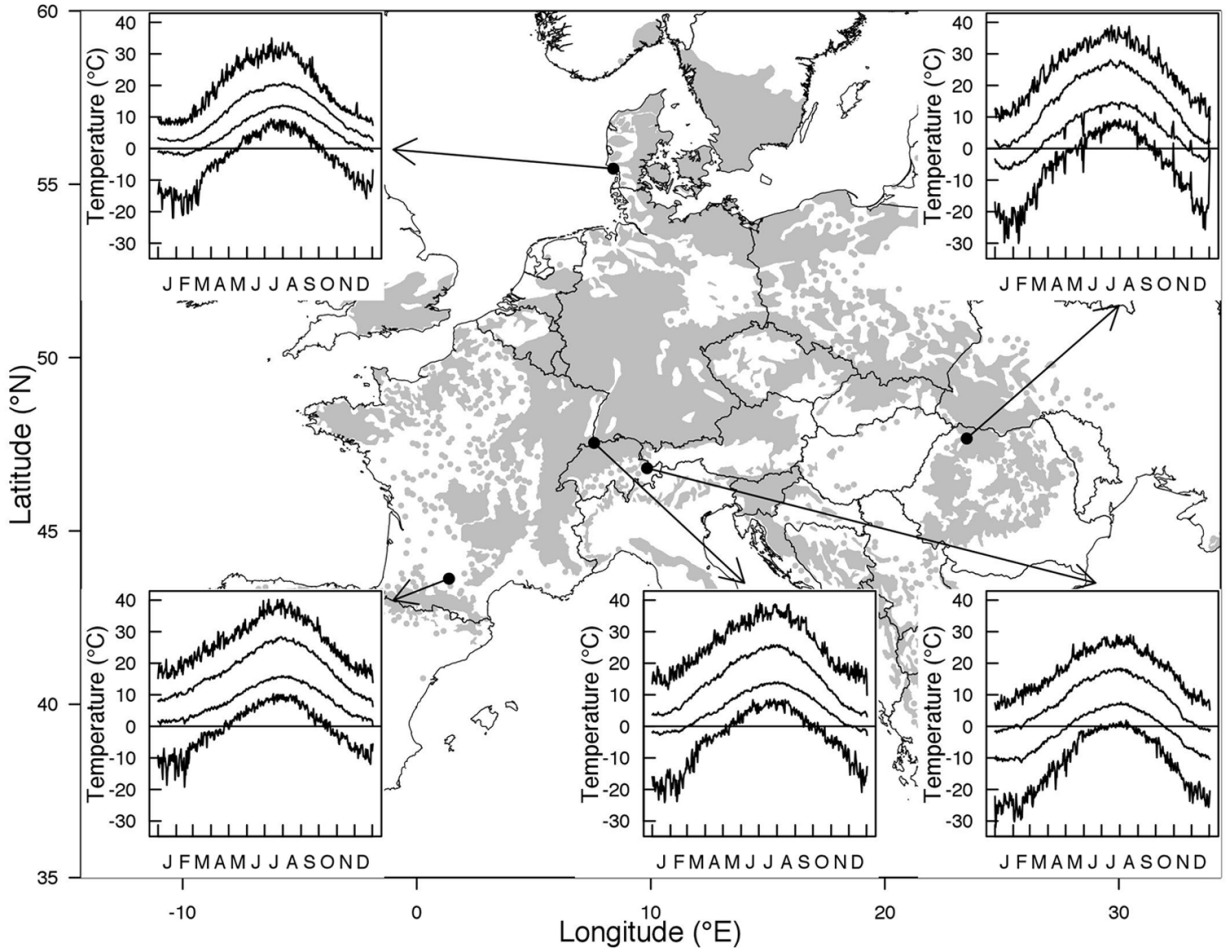
**Distribution range of *Fagus sylvatica* along with climatic characteristics at the northern and southern edge of the distribution and at low versus high elevation in central Europe.** Absolute maximum, mean maximum, mean minimum, and absolute minimum temperature for a given day of the year in the period 1901–2000 are shown. The selected stations were Toulouse (France, 43.6°N 1.4°E, 151 m a.s.l) in the West, Nordby (Denmark, 55.5°N 8.4°E, 4 m a.s.l) in the North, Baia Mare (Romania, 47.7°N, 23.5°E, 216 m a.s.l) in the East, Binningen (Switzerland, 47.5°N 7.6°E, 316 m a.s.l) at low elevation, and Davos (Switzerland, 46.8°N 9.8°E, 1594 m a.s.l) at high elevation.

Temperate climate generally refers to climatic conditions that prevail at latitudes between 40 and 60°. Temperate zones are characterized by large temperature oscillations at daily, monthly, and annual scale and a seasonal variation of photoperiod that increases with increasing latitude. While the photoperiodic signal is stable for any day of the year, temperature fluctuations are unpredictable. Within each of the four seasons large drops in temperature can occur from 1 day or 1 week to the next, yet within a seasonally distinct range. Furthermore, the contrast in temperature among the seasons increases with increasing continentality (distance from the coast). Trees in temperate regions must cope with freezing temperatures during any time of the year, especially from late autumn to early spring and in some exceptional years during early autumn or late spring. The freeze-free period is reduced toward northern latitudes and higher elevations as well as with the degree of continentality. For instance, *Fagus sylvatica* L., one of the most dominant tree species in Europe, experiences on average 320 freeze-free days near the south western edge of its distribution in France, less than 290 freeze-free days near its upper latitudinal distribution in Denmark or close to its eastern range limit in Romania, and about 166 freeze-free days at its upper elevational limit in the Alps (**Figure 1**, means calculated over the whole 20th century). However, this does not mean that the risk of freezing injuries for temperate trees increases with increasing latitude or elevation (Lenz et al., 2013), because the onset of the growing season shifts in parallel (see next section). In contrast to temperature, day length follows the same seasonal variation in a given location from year to year and thus, constitutes a reliable marker of the progression of the seasons.

### SHORT TERM TEMPERATURE FLUCTUATIONS WITHIN SEASONS

In temperate areas, it is relatively frequent that a cooler or a warmer mass of air causes abrupt changes in temperature within a 24 h period. While warm spells associated to severe drought can be highly critical for temperate trees in summer, they can also lead to a certain degree of dehardening in winter or early spring, which can be damaging in combination with a subsequent freeze event. Cold spells are potentially damaging in autumn and spring if the tissues are not hardy enough. Cold fronts move fast and can produce sharp decreases of temperature by rapidly replacing the warm (lighter) air. Cold spells can last from hours to days and are known to have caused food shortage and famines in the past centuries by killing crops before the harvest. In the northern hemisphere, the polar jet stream flowing west to east results from the difference between cold temperatures in the Arctic and warmer temperatures in the mid-latitudes and is strongly influencing the weather in the northern hemisphere at temperate latitudes. Recent studies suggest that the movement of the polar jet stream is changing with global warming because the northern polar regions warm much faster than the rest of the world (Semenov, 2012; Screen and Simmonds, 2013), which is amplified by a recent dramatic loss of artic ice and snow cover (Screen and Simmonds, 2010). Hence, the temperature difference between Arctic and mid latitudes shrinks and causes larger wavelength of the polar jet stream and weakens its flowing velocity (Francis et al., 2009). This has dramatic implications for climate stochasticity because the more the jet stream undulates north and south, the more changeable and extreme the weather is (Screen and Simmonds, 2013). Current Artic warming is therefore enhancing the frequency and magnitude of weather extremes in mid latitudes (Semenov, 2012).

### SPATIAL AND YEAR-TO-YEAR IN VARIABILITY IN TEMPERATURE AND LEAF-OUT DATES

**FIGURE 2:**
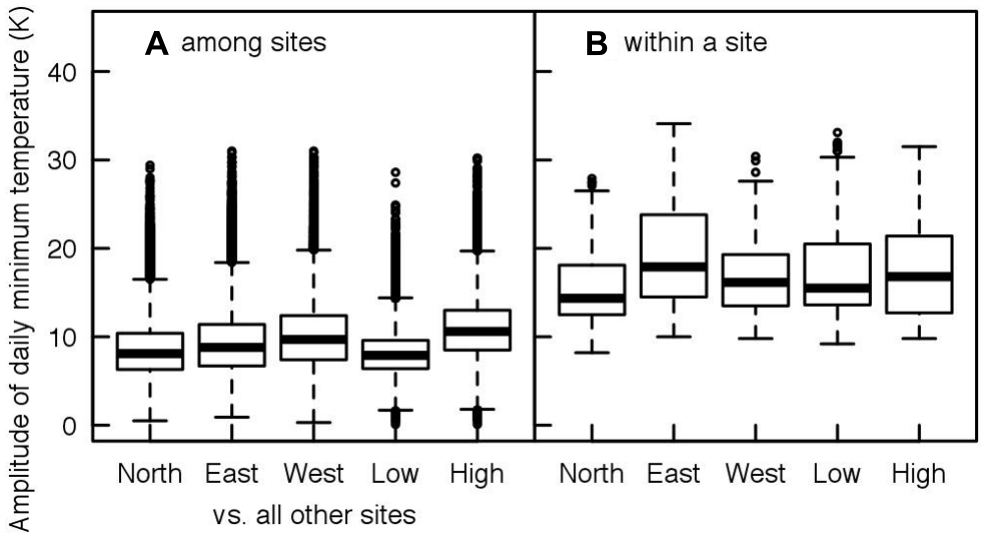
**Daily variation of minimum air temperature at five extreme locations within the distribution of *Fagus sylvatica* (see **Figure 1** for the location of the sites) for a same date among the five locations (**A**, spatial scale), and within a site for the same day of the year across years from 1900 to 2010 (**B**, temporal scale).** Note that the extent of temperature variation within a site for a given day exceed the maximum variation of temperature that occurs at a given date between the two extreme locations of the distribution area of the species.

**FIGURE 3:**
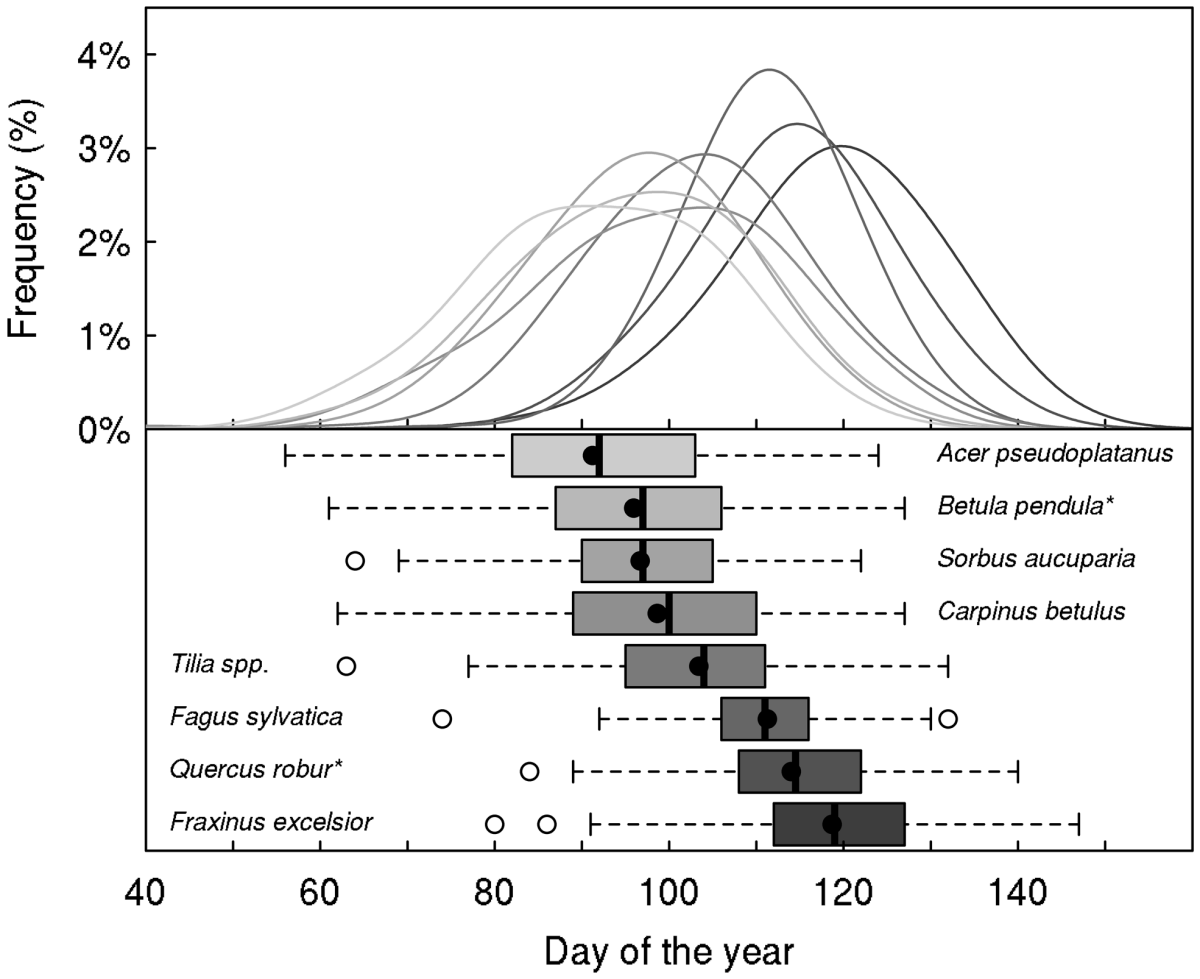
**Marsham phenological records (Sparks and Carey, 1995).** The upper panel shows the frequency of distribution of the leaf-out dates in Norfolk (UK) for eight temperate tree species over the period 1736–1958 (between 158 and 178 years available, depending on species). The distribution was fitted by a Gaussian–Kernel density distribution with a bandwidth of 7 days for each species. The box-plots show median, quartiles, and extremes of the phenological data, with the mean given as filled circle. Species names followed by an asterisk indicate that this is the probable species but we are uncertain.

Tree taxa in temperate regions experience a large variability in temperature during the year, across years but also across the distribution area of a species, as shown in **Figures 1 and 2** for *Fagus sylvatica*, one of the most dominant tree species in Europe. Interestingly, the variation in daily minimum temperature experienced by European beech is smaller among sites at the different limits of the distribution range than the inter-annual variation in temperature observed at a given location (**Figure 2**). The large variability in temperature experienced by a single tree in temperate areas among different years explains the annual fluctuations in the timing of leaf-out, flowering, and fruit ripening of temperate trees. For instance, an analysis in four tree species observed in the International Phenological Gardens established in Europe since the 1950s, Chmielewski and Rötzer (2002) reported variability in the beginning of the growing season of up to 25 days across Europe over the examined period of 30 years (1969–1998). A remarkable example is the analysis of the well-known Marsham phenological records from Norfolk, England (Sparks and Carey, 1995). This long-term series of phenological observations, gathering more than 150 years of observations, revealed a range of approximately 2 months (∼40–70 days) in the timing of leaf-out dates of major European tree species such as *Quercus robur*, *Fagus sylvatica*, *Acer pseudoplatanus*, *Fraxinus excelsior*, *Sorbus aucuparia*, *Tilia spp*., *Carpinus betulus,* or *Betula pendula* (**Figure 3**). The analysis of this unique long-term series shows that the occurrence of leaf-out dates follows a Gaussian distribution indicating that early springs occurred as frequent as late springs over this period (**Figure 3**). *Fagus sylvatica* is known as one of the most sensitive tree species to photoperiod in its spring phenology in Europe (Vitasse and Basler, 2013). Noteworthy, it is also the species that exhibited the lowest variation in its leaf-out timing in the Marsham phenological records (**Figure 3**).

When variability in flushing date is discussed, it is crucial to separate spatial (e.g., high vs. low elevation) from temporal (year to year) variation, and variation within a genotype vs. different genotypes. Further it is key to separate tree species that belong to different life history strategies (early successional, short lived, r-strategists vs. late successional, long-lived, k-strategists), trees that grow in the wild versus trees planted in cities or aboreta (provenances of unknown origin), and wild trees *in situ* vs. cultivated trees or exotic trees. Further, seedling or sapling responses must be separated from adult tree responses since they are known to operate under different phenology control than adults (Vitasse, 2013). Finally it makes a large difference if oceanic locations with mild winters and hardly any significant frost regimes are compared with continental climates. Variability in phenology across such vastly different plant types and life conditions do not permit generalization (theory development). The variability that matters is that within a given adult genotype at a given location and we lack such long-term phenological series.

## LONG-TERM EVOLUTIONARY RESPONSES OF TREES TO SEASONALITY

In the northern hemisphere, the seasonal climate of temperate regions has led winter deciduous trees to adapt by a dormancy period in winter, with no active growth, a low metabolic activity and a high tolerance against freezing temperature. In the following sections we will focus on the dormancy period and the environmental cues that trigger and release trees from this physiological stage. Then, we will discuss the structural and physiological adjustments during the dormancy period required to achieve a high degree of freezing tolerance. Finally, we will discuss the long-term genetic adaptation in cold hardiness among different tree populations inhabiting contrasting climatic conditions.

### ENVIRONMENTAL CUES INVOLVED IN THE REGULATION OF DORMANCY, ACCLIMATION AND DEACCLIMATION

The regulation and the maintenance of bud dormancy are highly complex as it involves the interaction of internal and external factors. For instance, it is assumed that the dormancy of most temperate tree species is released after they have passed through a certain duration of chilling temperatures between 0 and 5°C (Coville, 1920) which interact to variable degree depending on species with an increasing photoperiod (Cannell and Smith, 1986; Heide, 1993; Caffarra and Donnelly, 2011; Vitasse and Basler, 2013; Basler and Körner, 2014; Laube et al., 2014; Polgar et al., 2014). Chilling requirement and photoperiod controls allow temperate trees to avoid a too early development (in the case of warm winter weather) in relation to the probability of freezing. Longer durations of chilling and longer photoperiod decrease the thermal time required for budburst, i.e., decrease the warmth requirement for budburst (Sarvas, 1972; Basler and Körner, 2014; Dantec et al., 2014; Laube et al., 2014). This non-linear relationship between photoperiod and temperature may reflect the non-linear increase in spring temperatures along with the decrease in probability of freezing temperatures (Cannell, 1997) and can be seen as the most prominent adaptation to escape frost injuries to developing leaves by the appropriate timing for flushing (Lenz et al., 2013).

Dormancy induction is considered as a prerequisite to cold acclimation during the hardening period (Weiser, 1970; Pagter and Arora, 2013). It is widely recognized that the shortening of the photoperiod in combination with lower temperatures in autumn initiate and strengthen cold acclimation (e.g., Till, 1956; Weiser, 1970; Flint, 1972; Christersson, 1978; Arora et al., 2003; Beck et al., 2004; Kalberer et al., 2006).

Decreasing photoperiod and temperature cause active tissues to gradually enter into a dormant state (Welling et al., 2002), called hereafter endodormancy (Lang et al., 1987). During this process overwintering tissues harden to reach a maximum cold hardiness in mid-winter, and cells are then in a physiological state that allows them to withstand very cold temperatures. Under actual winter conditions, deciduous broad-leaved trees generally exhibit freezing resistance ranging from –15 to –25°C in roots, from –25 to < –35°C in shoot buds and from –30 to –50°C in twigs and stems (Till, 1956; Flint, 1972; Larcher, 2005; Körner, 2012). Nordic *Betula* species and other cold adapted species were found to tolerate temperature lower than –70°C in full dormancy (Sakai and Larcher, 1987; Körner, 2012). After an artificial hardening, freezing resistance of trees in winter can be further enhanced. For instance, twigs of *Salix pentandra* L. or *Morus alba* L. were shown to tolerate the temperature of liquid nitrogen (-196°C) when artificially hardened (Sakai, 1960; Stushnoff and Junttila, 1986).

In late winter and early spring, woody temperate species undergo two forms of deacclimation (Kalberer et al., 2006). “Active deacclimation” refers to a loss of cold hardiness as part of a general enhancement of metabolism and development in response to the combined effect of past chilling experience, photoperiod, and concurrent temperatures. Active deacclimation by temperature (forcing) typically comes into play during the spring-ecodormancy stage, once chilling and photoperiod requirements are fulfilled. In contrast, “passive deacclimation” of freezing tolerance results from the exposure of fully acclimated (hardy) trees in mid winter to small or moderate increases of temperature. Long days were shown to enhance deacclimation-associated growth and reduce reacclimation ability at specific developmental stages in *Pinus sylvestris* (Leinonen et al., 1997). Recently, Basler and Körner (2014) showed that the onset and duration of bud growth are co-controlled by photoperiod and temperature in cuttings of *Fagus sylvatica* and *Picea abies* and to a lower extent in *Quercus petraea*. However, photoperiod presumably plays a role in deacclimation-associated growth only for tree species that are photoperiodic sensitive in their spring development (Species experimentally found to be photoperiodic sensitive species in spring listed in Way and Montgomery, 2014).

### ESCAPE, AVOIDANCE, AND TOLERANCE OF FREEZING TEMPERATURES

To cope with freezing temperatures, temperate tree species adopt three strategies: escape, avoidance, and tolerance (Levitt, 1972; Körner, 2012). First, temperate deciduous trees escape the effect of freezing temperature on leaves in winter, by shedding them in autumn (the stress is not present for these tissues). To escape freezing damage to young sensitive leaves in spring, trees can adjust the timing of budburst. Escape of freezing temperatures by adjusting spring phenology constitutes a prominent adaptation of trees in temperate regions. Thus, as previously mentioned, the timing of budburst reflects the combined effects of three main environmental cues (chilling, photoperiod, and warm temperatures) in a way that young leaves are rarely exposed to severe freeze events. Yet, the relative contribution of each cue and their interactions remain to be clearly deciphered (Basler and Körner, 2014; Laube et al., 2014).

Avoidance can be regarded as the minimization or the exclusion of the stress effect on a given tissue. Trees can avoid ice formation in xylem parenchyma cells and bud tissue by the so-called supercooling process, where water remains in the liquid phase at sub-zero temperature, even though more stable phases would exist (Supercooling process in plants reviewed by Wisniewski et al., 2014). The limit for homogenous ice nucleation of water, i.e., in the absence of any nucleating agents, is supposed to be around -39°C. Supercooling of water can occur in buds and xylem parenchyma cells anywhere from a few degrees below zero down to as much as -60°C (Wisniewski and Fuller, 1999; Körner, 2012). For supercooling to be possible, the presence of ice barriers is necessary to prevent the spread of ice into tissues with water in a supercooled state. Tannins – related to phenols – and flavonol glycosides have been reported to act as anti-nucleating agents inside xylem parenchyma cells, promoting deep supercooling (Kasuga et al., 2010; Kuwabara et al., 2013). The exact role of antifreeze proteins in promoting deep supercooling is still unclear. Freezing avoidance by deep supercooling has been evidenced mostly in xylem parenchyma cells, bud meristems and flower buds of woody species in winter, but little is known about its possible role in freezing avoidance in leaf buds of temperate trees during the ecodormancy phase (Wisniewski et al., 2014). The formation of ice barriers itself could be regarded as a further mechanism to avoid freezing damage since ice barriers constitute physical barriers against propagation of ice in plant tissue (Kuprian et al., 2014; Wisniewski et al., 2014). A further avoidance strategy for a given tissue is extra organ freezing, which can be observed to protect leaf primordia in many tree species (Sakai, 1982; Pearce, 2001).

The tolerance of extracellular ice formation (intracellular ice formation would inevitably lead to cell death) is achieved via physiological changes, many of which enhance the tolerance of cells to dehydration. Indeed, extracellular ice formation induces a dehydration of the cell. The water potential of ice is much lower than the one of liquid water. During extracellular ice formation, an osmotic gradient is established between the intracellular water and the extracellular ice. Consequently, water is diffusing or transported out of the cell to balance the osmotic gradient, and cells dehydrate. Lethal freezing is usually related to damages to cell membranes when the freeze-induced dehydration exceeds the dehydration tolerance of the cell (Steponkus, 1984). The first stage of cold acclimation is induced by shorter day length in autumn and causes the accumulation of starch and lipid reserves in the overwintering tissues. Then, the second stage is regulated by cold subzero temperatures which induce numerous physiological and ultrastructural changes in the cells. Among the most important changes are the synthesis of stress proteins and dehydrins, changes to membranes, including the reduction in membrane located carriers and changes in phospholipid composition, ultrastructural changes of the cell, increasing concentrations of proline and gamma aminobutyric acid (GABA), as well as the synthesis of polyamines, and changes in carbohydrate concentrations. Most of these molecular ultrastructural changes have been discovered and documented during the second half of the last century in herbaceous plants. For a more detailed review of physiological alterations during hardening, we refer to Weiser (1970), Sakai and Larcher (1987), Kalberer et al. (2006), Preston and Sandve (2013), and Wisniewski et al. (2014).

### GENETIC ADAPTATIONS

As long-lived organisms with a large distribution range, trees must cope with very contrasting climates. Many tree species exhibit long distance pollen and moderate seed dispersal abilities (Kremer et al., 2012; Gerber et al., 2014). It is thus not surprising that temperate trees exhibit a high phenological plasticity. (Kramer, 1995; Vitasse et al., 2010, 2013), in spite of clear genetic differentiations related to the climate of origin (Vitasse et al., 2009a; Alberto et al., 2013). As previously demonstrated, the temporal variation of temperature in a single site exceeds the variation of temperature among extreme locations of the distribution area of a given tree species (see **Figure 2**). Therefore the high phenological variation in response to temperature enables temperate trees to adjust their growth period to a large range of climatic conditions within the distribution range rather than to be locally adapted. For instance, by adjusting the timing of leaf-out to temperature and photoperiod along elevational gradients, trees avoid the risky period in terms of freezing temperature for sensitive tissues such as new leaves (Lenz et al., 2013), irrespective of their elevation of origin, though high-elevation provenances seem slightly less flexible (Vitasse et al., 2013). Nevertheless along climatic gradients, a convergence of genetic differentiation among populations has been documented for deciduous temperate trees both for the timing of bud set and bud burst and cold hardiness. Thus, when planted in common gardens, seedling populations of winter deciduous temperate species from high latitude or elevation – i.e., colder climate – generally exhibit later spring phenology (Engler, 1913; Burger, 1926; Langlet, 1971; Barnett and Farmer, 1980; Vitasse et al., 2009a, 2013; Alberto et al., 2011; Körner, 2012) and earlier budset in summer (reviewed in Alberto et al., 2013) than populations from warmer climate, following the natural *in situ* variation, i.e., showing a co-gradient variation. European beech is an exception as the opposite pattern for the timing of budburst has been documented in numerous studies, suggesting that for this species a short season restricts the fitness more than the risk of freeze damage does (Vitasse et al., 2013; Lenz et al., 2014).

The genetic and phenotypic clines in cold hardiness also exhibit a co-gradient variation: young tree populations from high latitude were found to be more resistant than southern populations in winter when planted in common gardens as well as when assessed directly *in situ* (Larsen, 1986; Li et al., 2002, 2003; Viveros-Viveros et al., 2009; Kreyling et al., 2012b; Kreyling et al., 2014). The genetic cline is more marked along latitudinal gradients, because photoperiod controls the growth cessation and subsequently the initiation of the cold acclimation period in most temperate tree species (Junttila and Kaurin, 1990). The magnitude and the speed of deacclimation may differ between ecotypes as a result of genetic and environmental interactions. For instance, northern ecotypes of *Betula pubescens* were found to deacclimate slower than those from more southern latitudes (Taulavuori et al., 2004). While a few studies reported genetic differentiation among populations grown in common gardens in winter (Li et al., 2003; Kreyling et al., 2014), to our knowledge, no study has yet investigated whether there is a genetic differentiation among population in their freezing resistance during the most sensitive phenological stage, i.e., during flushing. Additionally to genetic differentiation in phenology and freezing resistance, slower growth rate in populations of young trees originating from colder climates has been generally reported in a number of studies both for conifers (Engler, 1913; Langlet, 1971; Rehfeldt, 1994; Oleksyn et al., 1998; Saenz-Romero et al., 2006; Rweyongeza et al., 2007) and broadleaved tree species (Engler, 1913; Langlet, 1971; Premoli et al., 2007; Vitasse et al., 2009a, 2014b), whereas only weak genetic differentiation was found in leaf morphological traits among populations along elevational gradients (Bresson et al., 2011; Vitasse et al., 2014b). These findings could reflect a trade-off between growth rate and freezing resistance. Regarding competition for light or water availability, rapid gain in height seems advantageous in warmer climate, whereas populations growing in colder climates, that are usually more open, have evolved under natural selection for greater general robustness, including cold hardiness (Loehle, 1998; Körner, 2012). However the trade-off between growth allocation and physiological adaptations to cold conditions is currently challenged. Indeed, the lower biomass increment found in populations originating from colder environment when planted in common gardens could result from the fact that these populations ceased to grow at shorter photoperiod, as elegantly shown recently in *Salix* species (Savage and Cavender-Bares, 2013) and early on in *Cornus stolonifera* (Smithberg and Weiser, 1968).

Remarkably, temperate trees are able to rapidly adjust their cold hardiness to temperature fluctuations during the endodormancy phase and to a lower extent during the acclimation and deacclimation phases via molecular and cellular adjustments. This is the focus of the next section

## RAPID RESPONSES TO TEMPERATURE FLUCTUATIONS

In temperate climates, rapid reductions in temperature – within a few hours – are possible during almost any time of the year. To cope with such short term changes in temperature, temperate trees employ first, a generally high level of freezing resistance during any time of the year, when compared with long-term records of low temperature extremes for a given period of the year (Lenz et al., 2013), and second, they developed the ability to rapidly acclimate the degree of freezing tolerance.

### RAPID ACCLIMATION AND DEACCLIMATION

During endodormancy temperate trees exhibit their maximum freezing resistance. For shoot buds, it has been reported to range between -25 and < –35°C (Larcher, 2005). However this range reflects what is measured at a time *t*. Several studies have demonstrated that these values can substantially fluctuate if temperatures prior to the measurement are exceptionally cold or warm (Pisek and Schiessl, 1947; Sakai, 1966; Sakai and Larcher, 1987; Neuner et al., 1999). Similarly, artificial hardening or sub-zero acclimation reveals the potential of trees to increase the cold hardiness of their overwintering tissues. Using herbaceous plants as model, this rapid acclimation to subzero temperatures seem to involve numerous unknown and stress-related genes (Herman et al., 2006) but the genetic and molecular basis of rapid acclimation during sub-zero temperatures seem distinct from that of cold acclimation during the hardening period (Le et al., 2008). Pronounced acclimation (as well as deacclimation) of freezing resistance by 5–15°C° in response to slight sub-zero temperatures have been observed in two conifer species and two dwarf shrub species (Pisek and Schiessl, 1947). In the light of these observations it appears very unlikely that native temperate trees suffer from freezing injuries in winter and that their low temperature range limits are set by absolute minimum temperature in winter. Consistently, long-term absolute minimum temperatures at species’ low temperature range limits, recorded over more than eight decades, were found to be much warmer than the maximum freezing tolerance of any temperate tree species (Lenz et al., 2013; Kollas et al., 2014).

Deacclimation in late winter and early spring proceeds much faster than acclimation in autumn (Kalberer et al., 2006), which makes trees vulnerable when a warm spell is followed by an abrupt cold spell, especially in late winter and spring. For instance, 1 day of deacclimation (warm greenhouse) in winter reduced the freezing resistance of *Pyrus malus* by about 15°C, whereas three cold days are necessary to recover the hardiness (Howell and Weiser, 1970). The authors also pointed out that the reacclimation after a deacclimation can only partially recover the initial degree of cold hardiness and this discrepancy increases with the progress of spring. Thus, in contrast to winter, during and prior to flushing, tissues reacclimate poorly in response to cold temperature (Pisek and Schiessl, 1947; Kalberer et al., 2006). The closer to bud break, the less easily buds become rehardened. Once warm temperatures have initiated growth resumption in spring, deacclimation is no longer reversible, as was demonstrated in *Picea abies* (Leinonen et al., 1997). This is why late spring freezing events are highly critical and play a key role in defining the low temperature range limit of temperate trees (Lenz et al., 2013; Kollas et al., 2014).

No correlation was found among species or ecotypes between the rate of deacclimation and the maximum cold hardiness reached before the deacclimation took place, nor with the climate of origin (Kalberer et al., 2006). The rate of deacclimation might relate more to the degree of temperature fluctuations to which trees are exposed rather than to low temperature *per se*. In other words, evolutionary pressure might have diminished the deacclimation potential during dormancy for species or populations that evolved in locations with frequent rapid fluctuations of temperatures in winter, while species or populations inhabiting more stable climates may be more sensitive to deacclimation in response to warm winter temperatures (Ogren, 2001). This is a field to be further explored given it is not known what fluctuation it needs for deacclimation to occur (Kalberer et al., 2006). Temperature fluctuations may also affect the timing of leaf-out as recently suggested by Kalvāns et al. (2014) and Wang et al. (2014).

Thus, the actual freezing resistance of a given tissue depends on the general hardening stage, plus the degree of short-term hardening in response to the recent history of temperatures. While the potential for acclimation is large in autumn and winter, it dramatically decreases over the course of spring. In contrast, the potential for deacclimation is low in late autumn and winter and exponentially increases in spring.

### RECOVERING FROM XYLEM EMBOLISM IN WINTER

Another consequence of the winter season in temperate areas is the winter embolism of the xylem that occurs in most temperate angiosperms. The embolism of xylem in winter is induced by freeze-thaw cycles (Sperry and Sullivan, 1992). When water freezes, the dissolved air gasses, causing air bubbles in conduits as the ice thaws (the “thaw-expansion hypothesis,” as for instance demonstrated in *Pinus contorta* by Mayr and Sperry, 2010). However, there is no evidence of lasting conduit failure, hydraulic continuity is seemingly re-established. At the least, the formation of new vessels in spring restores hydraulic conductivity, which is crucial in ring porous taxa which produce new vessels before bud burst (e.g., in *Quercus sp*., Cochard and Tyree, 1990), while others (diffuse porous) do so after bud burst (e.g., *Fagus sylvatica*, Cochard et al., 2001).

## CLIMATE CHANGE AND THE RISK OF FREEZE DAMAGE

### THE MOST RISKY PERIOD TO ENCOUNTER FREEZE INJURIES

Growth cessation and the onset of hardening in autumn are triggered by decreasing day length and cold temperature. Most tree species start hardening well before the first occurrence of freezing temperatures (**Figure 1**). For instance, an absolute minimum temperature of -14°C will occur at the coldest range edge of *Fagus sylvatica* only once every 100 years (Kollas et al., 2014). However, the LT_50_ values (median lethal freezing temperature) of *Fagus sylvatica* are already below -15°C in October for an average year (Tranquillini and Plank, 1989), and we expect them to be even lower in an extremely cold year due to the fast sub-zero acclimation. Similarly, modeling studies confirmed a much higher risk for freeze damage in *Picea sitchensis* growing in Scotland in spring than in autumn (Cannell et al., 1985). Summarizing, we suggest that the hardening period in autumn does not constitute a critical phase in terms of the risk to encounter freeze injuries. However an unusual early freeze event in autumn may lead to leaf abscission before leaves are fully senescent. Leaf senescence permits a remobilization of leaf nutrients and an early loss of leaves can affect carbon and nutrient storage (Norby et al., 2003) and the initiation and progression of acclimation (Charrier and Ameglio, 2011). Yet, some species take the risk and assimilate till leaves are damaged by freezing temperatures (N_2_-fixing taxa in *Alnus*, *Robinia,* and some *Salix* species; Tateno, 2003). As for autumn, to our knowledge little, if any, winter freezing injuries have been reported in native trees within their natural ranges in response to an abrupt drop of temperatures because the freezing resistance of overwintering tissues is far higher than the absolute minimum temperatures they encounter, except perhaps in *Picea rubens* Sarg. (DeHayes, 1992).

Among the different overwintering tissues, buds are of high importance because they contain leaf primordia and inflorescences, as well as apical meristems. In spring, after the release of endodormancy, buds enter the ecodormancy phase in which they de-harden stepwise and metabolism resumes. During that stage, buds gradually rehydrate and become more sensitive to freezing temperatures, until they reach a minimum in freezing resistance during leaf emergence (Neuner, 2007). The most sensitive phenological stage occurs during bud burst and leaf emergence, when cells are extremely active. During this stage LT_50_ values of temperate trees range between -2 and -8°C (Taschler et al., 2004; Neuner, 2007; Lenz et al., 2013) and are highly correlated with the date of flushing in mild and cold climates. Interestingly, new leaves of early flushing species are more freeze resistant than new leaves of late flushing species (Sakai and Larcher, 1987; Lenz et al., 2013; Vitasse et al., 2014a). In contrasts to autumn and winter, several late spring freeze events caused substantial damage to temperate forests in North America (Gu et al., 2008; Augspurger, 2009; Hufkens et al., 2012) and in Europe (Ningre and Colin, 2007; Kreyling et al., 2012a). For instance in 2007, all of Canada and most of the United States underwent a freeze after an abnormally warm period in late March and early April that triggered early leaf-out. Destruction of foliage due to a late spring freeze is a considerable loss for deciduous trees, since it negatively affects the tree’s nutrient pool, growth, reproduction, and canopy development. Yet, survival is commonly not affected, except if such events become too frequent. Freeze damage in spring can be partially compensated for by re-foliation, either from activation of dormant buds or development of adventitious buds. However, re-foliation requires additional resources and, most important, leads to a shorter growing season. For instance, after the late spring freeze in North America in 2007, trees recovered their canopy only after 16–34 days, and most of them showed only partial re-foliation. Further investigations need to be conducted to quantify the real impact of foliage destruction by late frost and at which frequency a foliage loss would cause serious damages to the tree. Experimental studies conducted *in situ* by defoliating parts or the whole canopy of trees to simulate the effect of herbivory may provide insights on the actual cost of foliage loss (e.g., Piper and Fajardo, 2014). It is likely that the loss of foliage due to a late spring freeze would have a larger impact on populations growing near the cold range boundaries, because they experience a very short growing season that restricts the remaining time for recovering or new shoots to mature. Because the end of the growing season is much less flexible than the beginning, given its photoperiod control, there is little leeway to prolong tissue activity. Such massive damages due to late freeze events are quite rare and temperate trees are rarely leafing-out at the “wrong time.”

### PHENOLOGICAL SHIFTS OF TEMPERATE FORESTS INDUCED BY CLIMATE CHANGE: IS THE RISK OF FREEZE DAMAGE INCREASING?

Climate warming has led to substantial phenological shifts in temperate trees over the last three decades. Earlier leaf-out and onset of growth has been detected by both, direct observations (e.g., Sparks and Menzel, 2002; Menzel et al., 2006; Fu et al., 2014b) and remote sensing (e.g., Zhou et al., 2001; Zhao and Schwartz, 2003; Zhang et al., 2004; Fu et al., 2014b). However, the phenological response of trees to climate warming is not linear due to interactions with chilling and photoperiod. Thus, the phenological shift is diminished with an increasing level of climatic warming (Körner, 2006), as was recently demonstrated by experimentally warming saplings of *Fagus sylvatica* and *Quercus* species (Morin et al., 2010; Fu et al., 2013). Less attention has been paid to the impact of climate change on growth cessation and leaf senescence (Estrella and Menzel, 2006), likely because the autumnal trend of cessation of cambial activity is photoperiodically controlled in most temperate tree species (Way, 2011; Takahashi and Koike, 2014). However, in some temperate tree species, such as *Sorbus aucuparia* or *Populus sp.,* temperature additionally regulates the timing of bud set and growth cessation (Perry, 1971; Heide, 2011; Rohde et al., 2011). Observations and satellite data suggest that climate change is going to extend the canopy duration of temperate trees in Europe, Eurasia, and North America in response to warming (Menzel and Fabian, 1999; Zhou et al., 2001; Delpierre et al., 2009; Vitasse et al., 2009b, 2011; Garonna et al., 2014; Jeong and Medvigy, 2014). While remote sensing is suitable to identify the onset of the growing season by canopy greening, it is hardly possible to identify the end of the active growth period with this method and the timing of leaf colouration has to be interpreted with caution. Leaf colouration might be a rather unsuitable symptom of physiological deactivation that is influenced by frosty nights, long after trees have gone through a cascade of hormonal and structural adjustment, including the formation of the abscission layer. Furthermore, the annual phenological cycle of temperate trees forms an integrated system with a shift in a given phenophase affecting the subsequent phenophase or even the timing of the next years phenophases (Heide, 2003; Fu et al., 2014a).

The occurrence of freezing temperatures in spring is very stochastic. Thus, spring phenology of temperate tree species has to be well adapted to escape freeze damages on longer time scales (Lenz et al., 2013). However, winters are expected to become progressively milder, with an increasing risk of exceptionally warm spells. Because warm spells may induce premature dehardening, the risk of subsequent freezing injuries in late winter and early spring could rise (Pagter and Arora, 2013). Since the mid 1980s, there has been an unresolved debate whether the risk of freeze damages in trees will increase in the next decades under continued climate change, with contrasting results. For instance, Hänninen (1991) predicted an increased risk of freeze damage under expected warmer climate scenario in Finland. Augspurger (2013) drew the same conclusion for 20 woody species in Illinois (USA), while no change or even a reduced risk was found in other studies in the Netherlands and Germany (Kramer, 1994; Scheifinger et al., 2003). These contradicting predictions root in the lack of data for sites where phenological observations were recorded along with air temperature over many years. In sites where such combined datasets are available, late spring freeze events are too rare to conclusively investigate whether the risk of freezing damage has changed over the last decades. Tree species that have low chilling requirements to break dormancy are at greater risk (Pagter and Arora, 2013). More data are needed on the potential of buds to re-acclimate in response to cold temperatures during the ecodormancy phase in spring (Kalberer et al., 2007; Pagter and Williams, 2011).

## CONCLUSION AND FUTURE DIRECTIONS IN PHENOLOGY RESEARCH

This review attempted to give an overview of the ecological significance of low temperature extremes in temperate trees. In general, temperate trees exhibit a sufficiently early onset of hardening in autumn to escape any damage from late season freezing temperatures. Further, trees are far more freeze resistant in winter than the minimum temperatures that have been recorded over centuries, and trees are able to rapidly harden in response to a rapid drop of temperature during this period. However, trees do also rapidly deharden during a warm spell in late winter or early spring. One of the current challenges is to address the extent to which tissues can reharden after a warm spell, and to predict how often these warm spells will occur in the near future.

The greatest risk for temperate trees to encounter freeze damages occurs during the period of leaf-out, though damages are only rarely observed under current climatic conditions. We emphasize that late spring freeze events are climatic extremes with high ecological and evolutionary importance, likely controlling the latitudinal and elevational range limits of temperate tree species (Sakai and Larcher, 1987; Lenz et al., 2013; Kollas et al., 2014). One of the main challenges today is to truly understand the effect of temperature and photoperiod on bud dormancy – from the initiation of endodormancy to budburst – as well as to quantify to what extent freezing resistance of buds varies in relation to temperature fluctuations, especially during the dehardening period, when buds are in ecodormancy. Spring phenology is the key to any risk assessment or prediction with regard to damaging low temperature extremes and the species range limits they set.

## AUTHOR CONTRIBUTIONS

Yann Vitasse led the writing with input from the two other authors.

## Conflict of Interest Statement

The authors declare that the research was conducted in the absence of any commercial or financial relationships that could be construed as a potential conflict of interest.
